# A Role for the Placenta in Programming Maternal Mood and Childhood Behavioural Disorders

**DOI:** 10.1111/jne.12373

**Published:** 2016-08-15

**Authors:** A. B. Janssen, D. A. Kertes, G. I. McNamara, E. C. Braithwaite, H. D. J. Creeth, V. I. Glover, R. M. John

**Affiliations:** ^1^ Cardiff School of BiosciencesCardiff UniversityCardiffWalesUK; ^2^ Department of Psychology and University of Florida Genetics InstituteUniversity of FloridaGainesvilleFLUSA; ^3^ Department of Experimental PsychologyUniversity of OxfordOxfordUK; ^4^ Faculty of MedicineImperial College LondonLondonUK

**Keywords:** maternal mood, prenatal stress, foetal programming, placenta, hormones

## Abstract

Substantial data demonstrate that the early‐life environment, including *in utero*, plays a key role in later life disease. In particular, maternal stress during pregnancy has been linked to adverse behavioural and emotional outcomes in children. Data from human cohort studies and experimental animal models suggest that modulation of the developing epigenome in the foetus by maternal stress may contribute to the foetal programming of disease. Here, we summarise insights gained from recent studies that may advance our understanding of the role of the placenta in mediating the association between maternal mood disorders and offspring outcomes. First, the placenta provides a record of exposures during pregnancy, as indicated by changes in the placental trancriptome and epigenome. Second, prenatal maternal mood may alter placental function to adversely impact foetal and child development. Finally, we discuss the less well established but interesting possibility that altered placental function, more specifically changes in placental hormones, may adversely affect maternal mood and later maternal behaviour, which can also have consequence for offspring well‐being.

## Introduction

Pregnancy can be a challenging time in a woman's life, during which she may experience many changes in her circumstances. Pregnancy can also be a time of vulnerability to perinatal mental illnesses, including depression, stress and postnatal psychosis 1. In the UK, it is estimated that maternal mood disorders, either prenatally or in the immediate period after birth, affect approximately one in five women, and is therefore one of the most common conditions of pregnancy. These disorders affect women of all ethnicities, nationalities and social strata 2. In addition to the distress that this causes women and their families, mental health disorders are one of the leading causes of death during pregnancy and the year after birth 1. Of equal concern is an additional consequence for her child's well‐being, with an increased risk of adverse behavioural and metabolic outcomes following exposure to maternal prenatal stress 3. Thus, a greater understanding of both the causes and the consequences of prenatal maternal stress and mood disorders is imperative.

## Foetal programming by maternal stress

Prenatal stress at its broadest level includes major life event stress, catastrophic disasters, chronic stress, daily hassles, perceived stress or pregnancy‐specific anxiety along with related symptoms of depression or general anxiety during pregnancy 4. Both animal and human studies indicate that maternal prenatal stress is associated with an increased risk of adverse emotional, behavioural and cognitive outcomes in the offspring, a subject that has been reviewed extensively 5, 6. Several studies, including the large Avon Longitudinal Study of Parents and Children (ALSPAC) cohort, allow for multiple confounders, including prenatal paternal and postnatal maternal mood. The findings from such studies indicate that the increased risk for adverse outcomes in the offspring is programmed *in utero* by the maternal emotional state, in least in part 7. If the mother is in the top 15% of a normal population for prenatal symptoms of anxiety or depression, her child has double the risk of a probable mental disorder, raised from approximately 6–12% at the age of 13 years 7. However, it is clear that not all children are affected equally by exposure to maternal stress, and also the effects of prenatal stress on child development are inconsistent. Recent data suggest that this may partly be a result of a gene/environment interplay, including the interaction between prenatal maternal anxiety and the child with respect to the genes for brain‐derived neurotrophic factor (*BDNF*) 8 and catechol‐O‐methyltransferase (*COMT*) (O'Donnell and Glover, unpublished data), during the development of emotional and cognitive outcomes, respectively.

## The role of the placenta in mediating foetal programming

**Figure 1:**
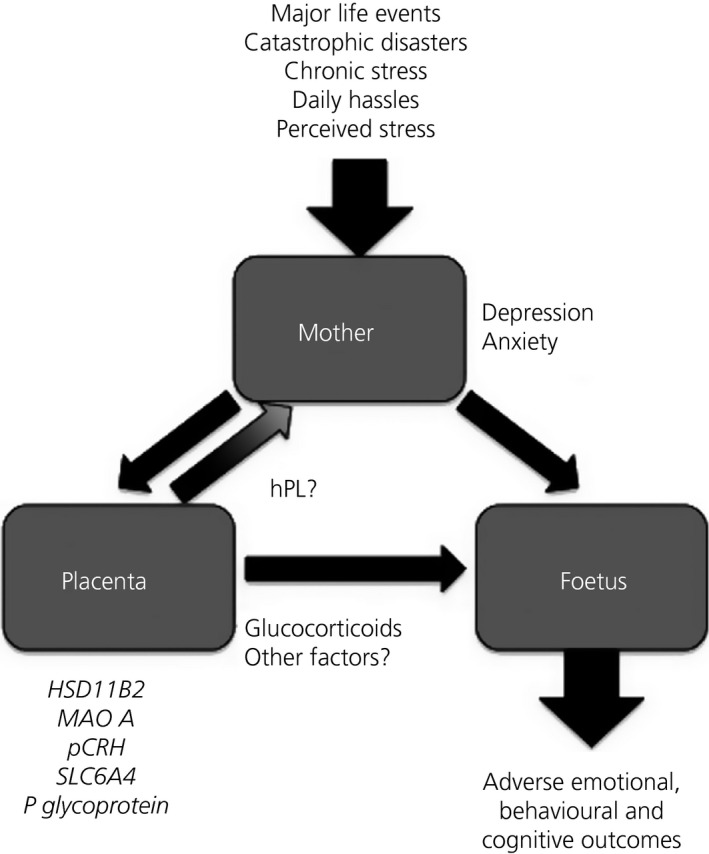
The role of the placenta in prenatal stress. Prenatal stress can influence the placental and foetal transcriptomes and epigenomes, which may contribute to adverse outcomes for children. Dysfunctional placental signalling may also influence maternal mood during pregnancy and maternal behaviour in the postnatal period, further contributing to adverse outcomes.

Placental function is important both for optimal foetal growth and maternal health 9. The placenta is a transient organ of pregnancy that transports nutrients and oxygen to the growing foetus and removes waste products. Additionally, the placenta functions to mitigate the mother's immune response to her semi‐allogeneic foetus and manufactures large quantities of hormones that flood the maternal system to induce the adaptations required for a successful pregnancy 10. An optimally functioning placenta can provide protection to the foetus against some forms of prenatal adversity. For example, the placental enzyme 11β‐hydroxysteroid dehydrogenase 2 (HSD11B2) regulates foetal exposure to maternal cortisol by converting it to inactive cortisone. Thus, the placenta is able to partially protect the foetus against elevated maternal cortisol levels. However, there is evidence that placental function may be affected by prenatal stress 11, 12, 13, 14, 15. Both endogenous and synthetic glucocorticoids have been shown to impact a variety of placental functions, including vascularisation, apoptosis and nutrient transport, in a range of animal models and human studies 16, 17, 18, 19, 20. Moreover, there is evidence that other maternal factors, such as catecholamines 21, may transfer the effects of maternal stress to the foetus by altering placental function. Consequently, suboptimal placental function induced by the maternal state may contribute to altered and, in our environment, poorer outcomes for children 22. Prenatal anxiety is associated with both lower expression and activity of placental *HSD11B2* 14, potentially mitigating the protective role that this enzyme usually plays, and theoretically exposing the foetus to higher cortisol levels. Foetal glucocorticoid exposure is also regulated by the placental glucocorticoid (GR; NR3C1) and mineralocorticoid (MR; NR3C2) receptors. Placental *NR3C1* and *NR3C2* expression is higher among depressed compared to nondepressed new mothers, providing another mechanism for the programming of adverse offspring outcomes 15. Alterations in the expression of genes for placental corticotrophin‐releasing hormone [*pCRH*; which stimulates the production of cortisol via the hypothalamic‐pituitary‐adrenal (HPA) axis], monoamine oxidase A (*MAOA*; which metabolises serotonin into 5‐hydroxyindoleacetic acid) and placental serotonin transporter (*SLC6A4*; which transports the neurotransmitter serotonin) and P‐glycoprotein have also been linked to prenatal stress 11, 23, 24, 25. Interestingly, very recent evidence suggests that the associations between prenatal maternal mood and placental gene expression may be different in Caucasian and non‐Caucasian populations (Capron and Glover, unpublished data). Thus, alterations in the expression of a number of genes in the placenta may mediate aspects of foetal programming associated with prenatal stress (Fig. 1); however, these findings may have ethnic specificities.

## Maternal stress and epigenetic changes in the placenta

Although it is clear that there are changes in the expression of genes in the placenta in relation to prenatal stress exposure, the mechanisms underpinning these changes are unclear. One possibility is the epigenetic deregulation of gene expression. Epigenetics describes the marks or tags that are added or removed from DNA sequence and the histones that regulate gene expression in a manner heritable through cell division. Such marks may be altered by exposure to stress during prenatal development, a period when the epigenome is rapidly changing 26. Animal research demonstrates that maternal stress can induce epigenetic changes in the placenta. DNA methylation changes have been reported in the promoter region of *Hsd11B2* 12 and altered chromatin methylation has been linked to the gene for O‐linked‐*N*‐acetylglucosamine transferase (*Ogt*) 27. Such alterations may be mediated by sex‐specific changes in the placental epigenetic machinery 28. In humans, maternal anxiety has been linked to greater placental methylation of *HSD11B2,* whereas maternal depression (but not anxiety) has been associated with increased placental methylation of *NR3C1* 29. Recent research with mothers in a conflict‐ridden region of the Democratic Republic of Congo has reported that higher levels of war trauma and chronic stress were associated with DNA methylation levels in multiple placental genes involved in HPA axis regulation, including *CRH*,* CRHBP*,* NR3C1* and *FKBP5* 30. Stress‐linked variation in DNA methylation was observed in placental tissue, as well as maternal and umbilical cord blood. However, similar to Jensen Pena *et al*. (12), the effects at individual CpG sites differed between tissues. The majority of CpG sites identified were situated in transcription factor binding regions, and several were associated with offspring birth weight 30. Similar effects of maternal war trauma and chronic stress have been observed with placental *BDNF* methylation 31. Considering that many affected CpG sites bind transcription factors, as well as the associations with mRNA levels or offspring birth weight, this suggests that stress‐linked variation in placental methylation may have functional consequences for offspring outcomes.

## Maternal stress and epigenetic changes in offspring

Experimental animal models have demonstrated that early‐life stress can leave a mark on the offspring epigenome, with alterations reported in offspring exposed to prenatal stress 28, maternal separation 32 and low levels of maternal postnatal care 33. In humans, variation in CpG methylation in umbilical cord blood at *NR3C1* has been associated with prenatal maternal anxiety 34 and depressive symptoms 29, 35. Also in cord blood, CpG methylation at both *NR3C1* and *CRH* is associated with maternal experiences of war trauma and chronic stress 30. The *NR3C1* and *CRH* CpG sites identified in these studies are the same as those linked to pre‐eclampsia in cord blood 36 or prenatal exposure to inter‐partner violence in offspring venous blood 37. In animal models, methylation at the *NR3C1* sites affects NGFI‐A binding 38. These may be sites for which DNA methylation is particularly sensitive to several stress‐linked phenotypes. Prenatal maternal chronic stress and war trauma is associated with *BDNF* methylation in cord blood at birth 31 and prenatal depression has been associated with DNA methylation at the *BDNF* promoter region in buccal cells at 2 months of age 39. BDNF plays an essential role in brain development and has been linked to psychiatric risk 40, thus suggesting that *BDNF* DNA methylation may be an important target for future investigation. Maternal cortisol and self‐reported depressive and anxiety symptoms have also been associated with altered DNA methylation of the imprinted genes *IGF2* and *GNASXL* 41. However, it is important to note that, across both rodent and human studies, there are tissue specific associations of prenatal stress and DNA methylation. These differences may reflect the different physiological functions of each tissue or differences in the epigenetic status of certain tissues at the time of exposure. Nonetheless, it is clear that the maternal stress can impact both the placental and the foetal epigenomes to alter gene expression.

## The programming of maternal mood by the placenta

Although considerable data from both animal models and human studies support changes in the placenta and foetus in response to maternal stress, which may then contribute to the programming of neurodevelopmental changes in offspring, few studies have explored a placental origin for maternal mood disorders. Pituitary prolactin and the placental lactogens are a group of evolutionarily and functionally‐related hormones important in pregnancy. Human placental lactogen (hPL) is produced by the placental syncytiotrophoblast and secreted into the maternal circulation, replacing prolactin as the main lactogenic hormone during pregnancy 42. Numerous studies highlight a functional role for these hormones and their shared receptor (prolactin receptor; PRLR) in the onset of maternal behaviours in rodents and, in the case of prolactin and PRLR, also maternal neurogenesis 43, 44, 45, 46, 47, 48, 49, 50, 51, 52, 53. In humans, these hormones may contribute to suppression of anxiety‐related behaviours during pregnancy 54. Decreased serum prolactin levels have been reported in human mothers with postnatal depression 55, 56, whereas increased levels of prolactin have been associated with low anxiety scores during pregnancy 57. Impaired hPL production has also been associated with adverse infant outcomes such as foetal growth restriction 58, 59. Thus, the altered placental expression of the genes for placental lactogen could contribute to both maternal mood disorders and adverse outcomes (Fig. 1).

## Imprinted genes, foetal programming and maternal mood disorders

Imprinted genes are expressed from one parental allele through epigenetic marking in the germline 60. Imprinted genes are known to regulate foetal growth, placental development, adult behaviour and metabolism 61. These multifunctional roles and the flexibility of epigenetic marks have led to the suggestion that imprinted genes may contribute to the foetal programming of adverse outcomes 62. Numerous studies have reported the aberrant expression of imprinted genes in the placenta in relation to foetal growth restriction and low birth weight 63. Altered expression of imprinted genes in the placenta has also been linked to infant neurobehavioral developmental outcomes 64, 65. Recently, imprinted genes have been highlighted as key regulators of the endocrine lineages that express placental hormones in rodents 66. This newly defined function suggests that the aberrant expression of imprinted genes in the placenta could contribute to the mispriming of maternal behaviour, at least in rodents, by modulating exposure of the maternal brain to key placental hormones such as the placental lactogens. If the function of imprinted genes in regulating the endocrine lineage was conserved across species, aberrant imprinting could help to explain the co‐occurrence of low birth weight with prenatal mood disorders, which has been reported in a number of studies 67, 68, 69, 70, 71, 72, 73. Epigenetic changes in cord blood DNA at imprinted loci have been associated with depressed maternal mood during pregnancy 74 and with maternal stress 41, 75. One study reported changes in DNA methylation in both cord blood DNA and placental DNA at an imprinted locus 76. Although it is generally presumed that such changes occur in response to prenatal adversity with the focus primarily concerning offspring outcomes, it is possible that such changes contribute to altered maternal mood by changing the endocrine function of the placenta (Fig. 1). Consistent with this hypothesis, recent data have identified a significant association between both symptoms of prenatal depression and clinically diagnosed depression with placental expression of both an imprinted gene and placental lactogen, a hormone predicted to be regulated by this gene 77. Determining the cause and effect relationships in human studies of pregnancy is challenging. However, very recent work utilising a novel rodent model suggests that imprinted genes in the placenta can influence both the maternal neural transcriptome during pregnancy and maternal behaviour in the early postnatal period (Creeth *et al*., in preparation), comprising data that support a causal relationship.

## Summary and outlook

In summary, the placenta plays a key role in supporting a successful healthy pregnancy. The placenta can provide a record of prenatal adversity, including maternal stress. Changes in placental function, potentially driven by epigenetic processes, may impact neurodevelopmental outcomes for children and both mental and metabolic health into adulthood. Importantly, placental dysfunction may also contribute to maternal mood disorders, either during pregnancy and/or in the immediate postnatal period, by exposing the mother to abnormal levels of placental hormones. At present, most women experiencing heightened levels of prenatal depression, stress and anxiety are undetected and untreated. The potential clinical implications of maternal prenatal mood on foetal and child neurodevelopment are substantial. The provision of better emotional care for all pregnant women and enhanced identification and support for women at particularly high risk of maternal mood disorders will help not only them, but also their children, and potentially subsequent generations.
